# Self-Reported Health, PERS, and Social Care Use in Older Adults at Risk of Falls: Pilot Results

**DOI:** 10.1093/geroni/igaf122.2837

**Published:** 2025-12-31

**Authors:** Nicole Zahradka, Jayer Chung, Anmol Momin, Alisha Williams, Ilse Torres Ruiz, Bijan Najafi

**Affiliations:** Best Buy Health, Boston, Massachusetts, United States; Baylor College of Medicine, Houston, Texas, United States; Baylor College of Medicine, Houston, Texas, United States; Best Buy Health, Boston, Massachusetts, United States; Baylor College of Medicine, Houston, Texas, United States; University of California, Los Angeles, Los Angeles, California, United States

## Abstract

Health-related quality of life (HRQoL) is a key outcome for aging in place interventions. Decreased HRQoL is associated with increased healthcare costs. This observational pilot study included 20 older adults (72±5 years old) at high-risk of falls who had access to a personal emergency response system (PERS) with fall detection, as well as 24/7 urgent response (UR) and social care (SC) services for 6 months. Outcomes were service utilization and change in self-reported HRQoL. Utilization was measured as a percentage of days the PERS pendant was worn over the study duration and percent of patients who engaged with UR and/or SC services. Self-reported HRQoL was measured with the Healthy Days instrument (CDC HRQOL-4) at baseline and monthly during study. The number of ‘unhealthy days’ and general health were compared between baseline and averaged monthly responses. Median days of pendant wear was 68.0 (56.1 – 84.4)% and 70% of participants engaged with at least one of the services: UR (n = 3), SC (n = 5), or both (n = 6). Mean unhealthy days at baseline was 18.0(20.0) days [physical=13.0(13.4) days, mental=5.0(9.7) days] and decreased to 14.7(16.8) days [physical=8.4(8.9) days, mental=6.1(8.8) days]. Average general health was rated as “good” at baseline and did not change. 12 out of 20 participants reported same or better general health during study compared to baseline. While there was an HRQoL improvement overall, within-subject changes varied. Aging in place solutions that address both urgent health status changes and social determinants of health have the potential to mitigate declining HRQoL associated with aging.

